# Validation of Selected MicroRNA Transcriptome Data in the Bovine Corpus Luteum during Early Pregnancy by RT-qPCR

**DOI:** 10.3390/cimb46070394

**Published:** 2024-06-27

**Authors:** Rreze M. Gecaj, Behlul Behluli, Curtis R. Youngs

**Affiliations:** 1 Department of Animal Biotechnology, Faculty of Agriculture and Veterinary, University of Pristina, 10000 Prishtina, Kosovo; rreze.gecaj@uni-pr.edu; 2 Department of Veterinary Medicine, Faculty of Agriculture and Veterinary, University of Prishtina, 10000 Pristina, Kosovo; 3 Department of Animal Science, Iowa State University, Ames, IA 50011, USA; cryoungs@iastate.edu

**Keywords:** microRNA, cattle, corpus luteum, corpus albicans, pregnancy, RT-qPCR, NGS

## Abstract

In cattle, the corpus luteum (CL) is pivotal in maintaining early pregnancy by secreting progesterone. To establish pregnancy, the conceptus produces interferon-τ, preventing luteolysis and initiating the transformation of the CL spurium into a CL verum. Although this transformation is tightly regulated, limited data are available on the expression of microRNAs (miRNAs) during and after this process. To address this gap, we re-analyzed previously published RNA-Seq data of CL from pregnant cows and regressed CL from non-pregnant cows. This analysis identified 44 differentially expressed miRNAs. From this pool, three miRNAs—bta-miR-222-3p, bta-miR-29c, and bta-miR-2411-3p—were randomly selected for relative quantification. Using bovine ovaries (n = 14) obtained from an abattoir, total RNA (including miRNAs) was extracted and converted to cDNA for RT-qPCR. The results revealed that bta-miR-222-3p was downregulated (*p* = 0.016) in pregnant females compared to non-pregnant cows with regressed CL. However, no differences in miRNA expression were observed between CL of pregnant and non-pregnant cows for bta-miR-29c (*p* > 0.32) or bta-miR-2411-3p (*p* > 0.60). In silico prediction approaches indicated that these miRNAs are involved in pathways regulating pregnancy maintenance, such as the VEGF- and FoxO-signaling pathways. Additionally, their biogenesis is regulated by GABPA and E2F4 transcription factors. The validation of selected miRNA expression in the CL during pregnancy by RT-qPCR provides novel insights that could potentially lead to the identification of biomarkers related to CL physiology and pregnancy outcome.

## 1. Introduction

With each new bovine estrous cycle, the ovary undergoes an extensive transformation and reorganization of its tissues, contributing to the complex process of reproduction [1]. The corpus luteum (CL), a transitory ovarian structure, undergoes a series of changes leading either to its maintenance and continuation of function (required for pregnancy) or to its regression and loss of functionality (luteolysis; required for a cow’s return to estrus and subsequent opportunity to ovulate and establish pregnancy). 

In early pregnancy, the developing embryo releases a signal (interferon-τ; INF-τ) to ensure the maternal recognition of pregnancy. This signal plays a critical role in preventing luteolysis and enables the continued secretion of progesterone by the CL. The adequate secretion of progesterone is essential for maintaining the early stages of pregnancy, as it supports the endometrium [2,3,4,5]. INF-τ contributes to CL maintenance by silencing *ESR1* expression. This silencing in turn prevents estradiol-induced oxytocin receptor expression and blocks the oxytocin-induced secretion of luteolytic pulses of prostaglandin F2α (PGF2α) [4]. These events, along with the process of angiogenesis and lymphogenesis, facilitate the sustained secretion of progesterone, thereby contributing to the prolonged life span of the CL and pregnancy [6].

The CL can spontaneously regress during four different time periods in cattle [5]. The first luteolytic period is near day 7 of the estrous cycle, leading to a short luteal phase and failure to establish pregnancy. The second luteolytic period occurs between days 18 and 25 as a result of repetitive prostaglandin F2α release by the uterus [5,7], and this typically manifests itself as a delayed return to estrus. The two other phases of CL regression occur during the early stages of pregnancy or in late pregnancy just before parturition [4].

Along with the complex hormonal control of gene expression in the regressing CL or the CL during pregnancy, there is growing evidence that post-transcriptional gene expression regulation complements the hierarchy of regulation events in the dynamic CL tissue [8]. A major class of non-coding single-stranded RNA molecules known as microRNAs (miRNAs) acts as regulators of gene expression at the post-transcription level by associating with the multi-protein RNA-induced silencing complex (RISC) [9,10]. The endogenous RISC pairs with the 3′-untranslated region (3′-UTR) of targeted mRNAs to fine-tune their expression [10,11], whereas the final mRNA expression is controlled by the mature 22-nucleotide-long miRNAs that are generated from their pre-miRNAs upon Dicer (RNase III enzyme) cleavage [12]. Once cleaved, the mature miRNAs are exported into the cytoplasm where they can up- and/or downregulate genes [12].

MicroRNAs can be secreted from tissues/cells via extracellular vesicles (EVs) and subsequently circulate stably in body fluids such as blood, milk, or urine [13]. Early pregnancy detection is vital for the efficient herd management of dairy cattle, and farmers strongly desire improved assays (e.g., qPCR quantification of miRNAs) for the earlier detection of pregnancy and/or infertility [14]. The endocrine nature of the corpus luteum (CL) implies its potential to secrete miRNAs that are pertinent to pregnancy physiology. The rigorous validation of potential biomarkers for pregnancy outcome across independent analysis platforms is essential to guarantee their reliability and accuracy. Once validated, these miRNAs could be easily sampled during routine animal milking procedures or veterinary check-ups and serve as a non-invasive sampling method for monitoring the reproductive status of dairy cattle. Indeed, there are published data suggesting that levels of specific miRNA such as miR-26a are increased in circulation as early as day 8, which is sooner than previously reported in any species [15]. Moreover, the utility of these biomarkers extends beyond veterinary applications, and the validation of miRNAs originating from the CL could also be useful in human reproductive medicine, offering new avenues for early pregnancy detection and therapeutic interventions [13,16].

In farm animals, the presence of miRNAs has been described in a wide range of different tissues [17]. In cattle, miRNAs have been identified by RNA-Seq in tissues including adipose tissue, skeletal muscle, oocytes, and the early embryo [18,19]. All three miRNAs investigated in our study—bta-miR-222-3p, bta-miR-29c, and bta-miR-2411-3p—are expressed in cattle. For example, bta-miR-222-3p, a member of the miR-221/222 family, modulates vascular function in late-cycle CL [20], and miR-29 regulates oxidative stress and inflammatory response in bovine mammary epithelial cells (bMECs) through various genes [21]. Bta-miR-2411-3p has been associated with the heat stress response observed in Frieswal (Holstein Friesian × Sahiwal) crossbred dairy cattle [22].

Very few studies, however, have explored the miRNA transcriptome in the CL of cattle [23,24,25]. One study identified the presence of miRNAs in the CL of pregnant buffalo by using RNA-Seq, which is a next-generation sequencing (NGS) technique [26]. Although a very good correlation between RNA-Seq and RT-qPCR data has been reported [27], discrepancies are reported when NGS results are validated by RT-qPCR [28]. In very dynamic tissues such as the CL, these discrepancies may be more pronounced.

This study aims to validate, using RT-qPCR, the RNA-Seq trend of the differential regulation of three randomly selected miRNAs (bta-miR-222-3p, bta-miR-29c, and bta-miR-2411-3p) in regressed CL versus CL during pregnancy. We hypothesize that the selected miRNAs that show a differential expression (as measured by the absolute quantification using RNA-Seq) will demonstrate the same trend of regulation and can be successfully validated by RT-qPCR.

## 2. Materials and Methods

### 2.1. Sample Collection

Bovine ovaries were collected at a commercial abattoir within 20 min of routine slaughter of animals presented by farmers. The reproductive status (pregnant, non-pregnant) of slaughtered cattle was determined by a morphological examination of the ovaries’ color and texture, as well as the evaluation of the consistency of the uterine mucus. Based on this comprehensive evaluation, the CL status of each animal from which CL were collected was categorized as previously described [25,29]. In the category of regressed CL, animals beyond day 17 of the estrous cycle were included, and in the category of CL during pregnancy, only animals within the first three months of gestation were considered. The gestational age was estimated by visual assessment of fetal size [30]. All ovaries were placed on ice for transport to the laboratory where they were dissected; excised CL were stored at −20 °C until RNA extraction. A total of 14 CL was studied, with n = 7 each for regressed CL and CL during pregnancy.

### 2.2. Total RNA Isolation and RNA Integrity Assessment

Total RNA was extracted from CL tissue and subjected to QIAzol extraction as previously described [25]. In brief, approximately 50 mg of CL tissue was homogenized with 700 μL of QIAzol for 50 s using a TissueLyser (Qiagen, Hilden, Germany) and cooled on ice. Centrifugation at 14,000× *g* separated debris. The clear supernatant was further processed by the miRNeasy Mini Kit (Qiagen, Hilden, Germany) for the isolation of total RNA (including miRNAs). The purity and concentration of RNA were measured on a BioPhotometer spectrophotometer (Eppendorf, Hamburg, Germany).

The integrity of isolated RNA was assessed using a Bioanalyzer 2100 (Agilent Technologies, Santa Clara, CA, USA), and the RNA integrity number (RIN) varied between 6.1 and 7.0. Although an RIN in that range is rather low for RT-qPCR, considering the fact that there is massive tissue degradation and/or reorganization occurring in the regressing CL and the CL during pregnancy [2,3], a phenomenon involving RNA degradation [31], this RIN number is considered acceptable.

### 2.3. Conversion of Total RNA to cDNA and RT-qPCR

Total RNA (100 ng) from 14 samples (7 regressed CL [also known as a corpus albicans {CA}] and 7 CL during pregnancy) was reverse transcribed using 5× miScript HiSpec buffer, transcriptase mix, 10× Nucleix mix, and H_2_O (miScript II RT Kit, Qiagen, Germany). Samples were incubated for 60 min at 37 °C and thereafter for 5 min at 95 °C.

Samples were diluted 10:100 with RNase-free water before initiation of PCR. Real-time PCR (RT-PCR) was performed using Rotor-Gene Q with the Rotor-Disk 72 system. The miScript PCR assays (Qiagen, Germany) were used for validation of the NGS results.

The miRNAs were quantified. For the in vitro amplification of miRNAs, the following miScript-PCR tests (Qiagen, Germany) were used: bt_mir-222_1, bt_mir-29c_1, and bt_mir-2411_1 targeting the mature miRNAs sequences (see Table 1). RNA pools lacking an RT enzyme from different CL samples, as well as additional negative controls, were included in each qPCR run. The expression level of miRNAs was assessed after their normalization against the Hs_RNU6-2_1 gene, and measurements were undertaken following the Pfaffl method [32].

### 2.4. In Silico Analysis

To predict the targets of the analyzed miRNAs, the microT-CDS tool from DIANA lab (https://diana.e-ce.uth.gr/tools (accessed on 18 November 2023)) was used [33]. The functional classification of miRNA hits was performed on Pantherdb (https://www.pantherdb.org/ (accessed on 18 November 2023)) [34]. A heat map of the significant miRNA signature for pathways was generated using miRPathDB v2.0 (https://mpd.bioinf.uni-sb.de/ (accessed on 19 November 2023)) [35].

### 2.5. RNA-Seq Data Re-Analysis and Statistical Analysis

To ascertain the differential regulation of miRNAs between pregnant and regressed CL, we conducted a re-analysis of previously published data from our group [36,37]. This involved statistical analysis of read counts using the R package DESeq2 from Bioconductor (https://bioconductor.org/packages/release/bioc/html/DESeq2.htmL (accessed on 8 August 2023)). This methodology relies on a negative binomial distribution, which establishes a connection between the mean and variance through local regression [38]. It calculates adjusted *p*-values (balance the rate of false positives or false discovery rate) using the Benjamini and Hochberg method, enabling comparison across samples. A cut-off of >50 read counts was used to identify differentially expressed (DE) miRNAs.

For RT-qPCR quantification, statistical analysis of data normalized against the Hs_RNU6-2_1 gene was performed using the Student’s *t*-test on dct for comparison between the regressed CL (i.e., CA) and CL during pregnancy. Means were considered statistically different at *p* < 0.05.

## 3. Results

**Figure 1:**
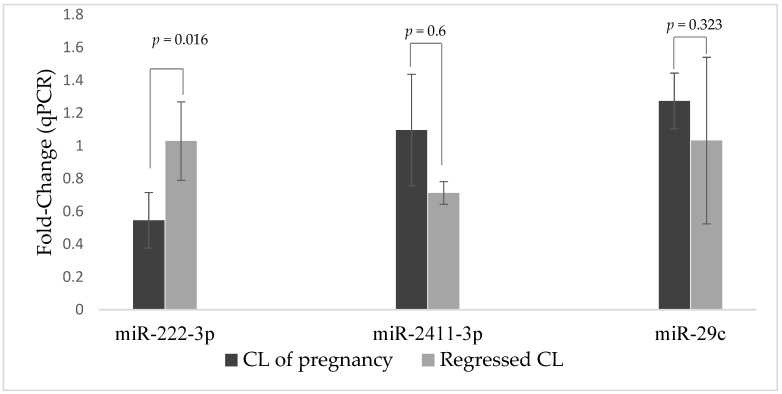
The expression of miRNAs in the pregnant corpus luteum (CL) regressed CL. The RT-qPCR fold change ratio was measured using the 2^−ddct^ from fourteen CL (seven from each group). Comparison of mean values was performed using Student’s *t*-test on the dct values, and the *p*-values for each pair-wise comparison are indicated for each miRNA. Means were considered statistically different at *p* < 0.05.

The expression of the three miRNAs (bta-miR-222-3p, bta-miR-2411-3p, and bta-miR-29c) in regressed CL (CA) and CL during pregnancy is depicted in Figure 1. The expression of bta-miR-222-3p was downregulated (*p* = 0.016) in the CL of pregnant females compared with regressed CL. On the other hand, the expression of miRNA in regressed CL and CL of pregnant animals was not different for either bta-miR-2411-3p (*p* = 0.60) or bta-miR-29c (*p* = 0.323).

The miRNA expression patterns identified through RT-qPCR in this study were consistent with those observed in the re-analysis of RNA-Seq data, as summarized in Table 2.

The number of read counts for bta-miR-222-3p is 152, and its expression is downregulated (−1.5, Table 2) in CL during pregnancy compared to regressed CL. The two other miRNAs, bta-miR-29c and bta-miR-2411-3p, show a sequencing frequency of 117 and 662, respectively. Both of these miRNAs are upregulated in the CL during pregnancy. Other miRNAs listed in Table 2 exhibit varying read counts, with bta-miR-150 and bta-miR-21-5p demonstrating the lowest and highest abundances, respectively.

Two miRNAs, bta-miR-3141 and bta-miR-2484, exhibit a fold change regulation greater than 2, whereas all other miRNAs show a fold change regulation less than 2 (Table 2).

**Figure 2:**
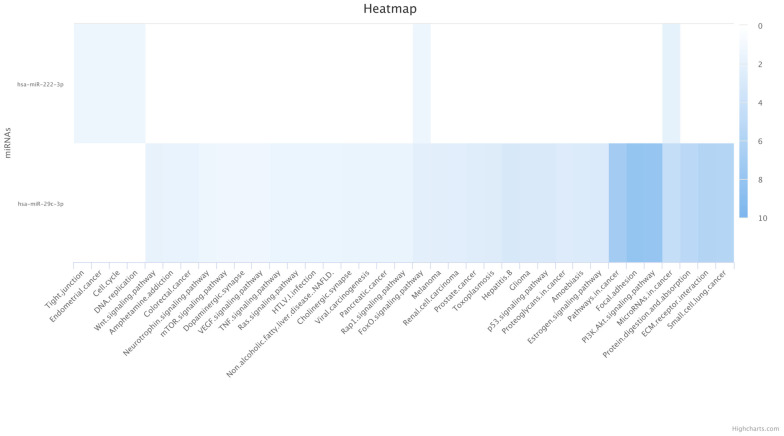
Enrichment results of hsa-miR-222-3p and hsa-miR-29c-3p for the categories of the KEGG database and experimentally validated miRNA–target interactions (MTIs). Rows: enrichment results for the targets of the two miRNAs; columns: all KEGG pathways that are significant for the different miRNAs. The absence of bta-miR-2411-3p on the heat map indicates that no enrichment was observed for this miRNA. The color of each field represents the significance of the association between the miRNA and the target pathway, depicted as the −log(*p*-value). Darker colors indicate more significant associations between the miRNA and the target pathway.

The enrichment results of bta-miR-222-3p and bta-miR-29 for the categories of the KEGG database and experimentally validated microRNA–target interactions (MTIs) are presented in Figure 2. The rows depict the enrichment results for the targets of these two miRNAs, while the columns enumerate all the KEGG pathways that demonstrate significance.

The analysis unveiled significant enrichment in pathways signaling the wingless-type MMTV integration site family (WNT), vascular endothelial growth factor (VEGF), forkhead box transcription factor (FOXO), and the extracellular matrix (ECM) (see Figure 2). Notably, only two categories, the FOXO pathway and microRNAs in cancer, were commonly significant for miRNAs (bta-miR-222-3p and bta-miR-29), with a *p*-value < 0.045. The pathway enrichment was conducted using the miRPathDB v2.0 (https://mpd.bioinf.uni-sb.de/ (accessed on 19 November 2023 and on 26 June 2024). However, for bta-miR-2411-3p, no enrichment was observed in the pathway databases.

**Figure 3:**
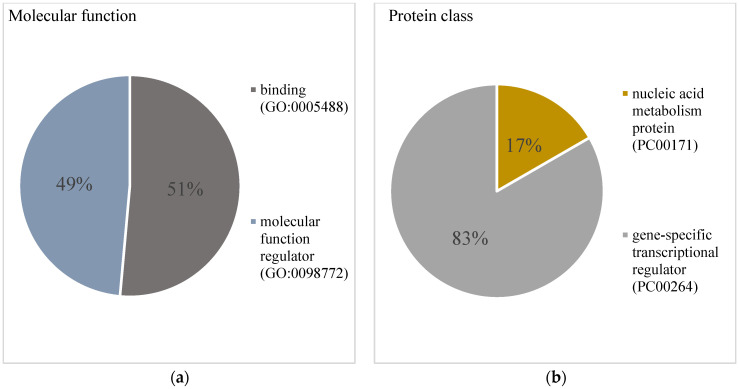
The molecular function and protein class gene ontology (GO). (**a**) For molecular function, the GO term “binding” represented 51% and the GO term “molecular function regulator” represented 49% of hits against the total number of genes. (**b**) For protein class, the GO terms “nucleic acid metabolism protein” and “gene-specific transcriptional regulator” represented 17% and 83%, respectively, of hits against the total number of genes.

The functional classification, carried out using Pantherdb (https://www.pantherdb.org/ (accessed on 18 November 2023), revealed two gene ontology (GO) terms within the molecular function ontology: binding (51%) and molecular function regulator (49%). Regarding the protein class, the analysis predicted the representation of nucleic acid metabolism protein and gene-specific transcriptional regulator GO terms with 83% and 17% of gene hits, respectively (Figure 3).

To elucidate the biogenesis regulation of the successfully validated bta-miR-222-3p, we utilized DIANA-miRGene ^V.4^ [39], a repository that links miRNA transcription start sites (TSSs) with their respective transcription factors. Employing a miRNA-oriented mode with ovarian tissue samples archived in the repository, we predicted two distinct transcription factors: the GA-binding protein (GABP) transcription factor alpha subunit (GABPA) and the E2 transcription factor 4 (E2F4). The search was conducted using default filter criteria for transcription-factor-binding sites (TFBSs) within the region, which defines the distance from the predicted miRNA transcription start site (TSS) of the upstream (3000 bp) and downstream (500 bp) boundaries. Additionally, default criteria for the *p*-value and expression threshold (tags per one million, TPM) of transcription factors were applied.

## 4. Discussion

MicroRNAs are post-transcriptional regulators of many physiological processes, including the regulation of pregnancy [25,40,41]. In cattle, miRNAs were profiled in plasma by sampling animals on the 8th, 16th, and 24th day of pregnancy [15]. The miRNA abundance was evaluated in milk by sampling on days 4, 12, and 18 after artificial insemination (AI) [28]. miRNAs have also been analyzed in the cells of the bovine corpus luteum (CL) 17 days after AI [24].

Using RT-qPCR, we showed that bta-miR-222-3p expression was downregulated in the CL of pregnant females compared to the expression observed in regressing CL. However, no differences in miRNA expression were found between CL of pregnant cows and cows with regressing CL for bta-miR-29c or bta-miR-2411-3p. In contrast, RNA-Seq data (Table 2) showed an upregulation of bta-miR-29c and bta-miR-2411-3p expression in CL during pregnancy compared with regressed CL. RT-qPCR did reveal that the expression of bta-miR-222-3p was downregulated, consistent with the results of the current study.

Using human luteinized granulosa cells (hLGCs), specific miRNAs (e.g., miR-96 that targets FOXO1) regulated luteal development by affecting cell survival and steroid production [42]. Because miRNAs are stable in the bloodstream in the form of extracellular vesicles (EVs), either as exosomes or microvesicles [13], they represent potential biomarkers not only for the early detection of pregnancy but also pregnancy complications such as pre-eclampsia and restricted intrauterine fetal growth [43].

Most of the data on miRNA expression during pregnancy were likely determined from samples of body fluids. However, miRNA assessments in tissues that directly support pregnancy (e.g., the CL) are required to better understand the physiology of pregnancy maintenance, especially during the first two months when pregnancy loss often occurs. Regardless of the biological sample source, for a miRNA to be classified as over- or under-expressed, the differential expression level should first be analyzed through a transcriptome sequencing methodology and then the data confirmed by using RT-qPCR [44]. RT-qPCR is typically performed to confirm whether transcripts are indeed significantly regulated in the same samples (technical replicates) or in biologically distinct samples (biological replicates) [45,46].

Although several studies have shown a very good correlation between RNA-Seq data and RT-qPCR data [47,48], different studies were unable to provide their full validation [25,28]. Full validation, using an independent approach, is essential if the miRNAs being investigated are intended for use as biomarkers of early pregnancy [28].

In one study aimed at identifying changes in circulating miRNA levels in early pregnant cows [49], only seven of fourteen selected miRNAs (50%) could be fully validated, while three of the fourteen miRNAs (21%) yielded differential expression in opposing directions. In our study, we also obtained similar results; we were able to validate, using independent analysis, only bta-miR-222-3, while no differences were observed for bta-miR-29c or bta-miR2411-3p. We randomly selected the miRNAs for validation, but we were not able to balance them in terms of read counts. Therefore, bta-miR-222-3p and bta-miR29c had a lower read number compared to bta-miR-2411-3p. Because all three miRNAs had the same differential expression in CL of pregnant cows compared with regressed CL, it is unlikely that the read count number had an impact on the successful validation of bta-miR-222-3p. In contrast, genes with low expression levels and a fold change >2 are considered the “non-concordant” fraction of genes, which typically also show a shorter expression time [44].

When measuring miRNAs at a single time point, the high natural inter-animal variations are another problem that potentially hinders miRNA expression validation and biomarker identification [28]. This obstacle is expected to be even greater considering the highly dynamic nature of the CL and the fact that we performed the validation on biological replicates. These factors can explain the discrepancies in validating all three selected miRNAs. In very dynamic tissues, it is essential to carefully define the time points and consider a higher number of animals in each group to reduce the variation due to sampling in the population being studied.

The miRNA successfully validated in our study (bta-miR-222-3p) is expressed in the bovine uterus and cumulus cells [23]. Cumulus cells (which originate as granulosa cells) are vitally important in ovarian follicular development and are closely linked with the oocyte by controlling its growth. While numerous cumulus cells are expelled from the ovarian follicle at the time of ovulation, some remain in the recently ruptured antral ovarian follicle and subsequently contribute to the formation of the CL (residual granulosa cells develop into large luteal cells of the bovine CL). While the involvement of cumulus cells in the functionality of the CL once it is fully formed is not known, it seems likely that granulosa cells are not as crucial for luteal function as they are for folliculogenesis [50].

Specifically, bta-miR-222-3p is a member of the miR-221/222 family, known for their similar sequences and consequent overlap in target genes. For instance, bta-miR-221 modulates vascular function in late-cycle females by targeting genes such as THBS1 and SERPINE1 [20]. Given the importance of maintaining established vasculature in the CL during pregnancy, both of these miRNAs could fine-tune the expression levels of other factors crucial for CL survival. In our study, we were not able to validate bta-miR-29c; however, other studies suggest it may regulate oxidative stress and inflammatory response in bovine mammary epithelial cells (bMECs) through various genes [21]. Notably, among others, it implicates forkhead box O1 (FOXO1), tumor necrosis factor-alpha (TNF-α), and major histocompatibility complex as potential targets influenced by miR-29c [21]. The available data on bta-miR-2411-3p are scarce and they suggest its involvement in heat stress response, as observed in Frieswal crossbred dairy cattle, known for their adaptation to environmental temperature stress [22]. Additionally, in a very recent paper, bta-miR-2411-3p was identified as an indicator of beef quality defects, particularly involved in cellular responses to redox imbalances and apoptosis [51].

**Figure 4:**
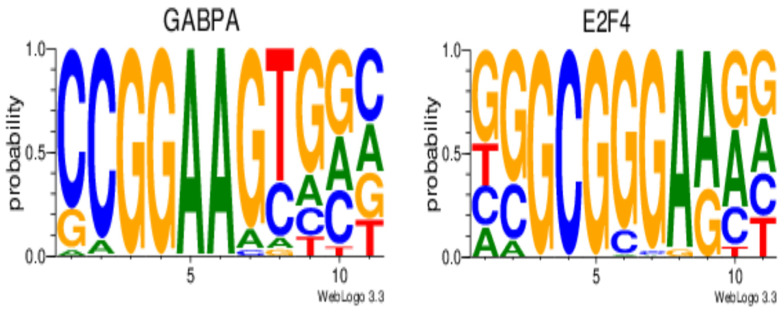
A graphical representation of binding sites for the transcription factors (TFs) overlapping transcription start sites (TSSs) of miR-222 within chromosome X as predicted by DIANA-miRGen ^V4^.

Next, we used an in silico approach to identify the transcription factor motifs within the promoter of the miR-222-3p gene. Transcription factors (TFs) are important proteins that regulate the transcription of specific genes, including miRNA genes, and are crucial in the process of gene expression [52]. Binding sites for TFs (GABPA and E2F4) were identified in this study (Figure 4). Previous research suggests that GABPA plays a regulatory role in controlling DICER1 by binding to the DICER1 promoter [53]. DICER1 is a pivotal enzyme in the process of miRNA maturation, and disruptions in the regulation of this enzyme can lead to significant downstream consequences [53]. The E2F transcription factors are essential for controlling the timing of gene expression during the cell cycle, including genes that oscillate in activity [54]. They also play a key role in regulating the expression of non-coding RNAs, including miRNAs [54]. However, whether or not E2F4 is involved in the regulation of bta-miR-222-3p expression needs to be validated by an in vitro follow-up experiment.

We showed that bta-miR-222-3p and bta-miR-29c are enriched in two pathways: FOXO and microRNAs in cancer. Numerous studies have associated miR-222-3p with cancer cell proliferation and apoptosis, as well as drug resistance via the suppression of FOXP2 [55,56,57]. These previous findings align well with our results because cell proliferation and apoptosis are processes that characterize CL development and regression [3]. In particular, apoptosis is induced in large luteal cells to cause CL death. In accordance with our findings regarding the upregulation of bta-miR-29c in the CL during pregnancy, a study aimed at deciphering the miRNA network at the maternal–fetal interface in pigs showed that miR-29c was maternally expressed [58]. Another pathway, WNT, is significantly enriched with gene targets of bta-miR-29c and involved in CL maturation [59,60].

Our analysis of the gene ontology terms enriched with genes targeted by the miRNAs we studied showed that 83% of those genes function as specific transcriptional regulators; 51% are active in molecular binding.

The expression validation of the selected miRNAs in the CL from pregnant females provides new data. These data can potentially be used to identify other biomarkers that are related not only to CL physiology and pregnancy outcome but also to animal reproduction in general, as well as other physiological processes that occur through the same signaling pathways. Additional research, however, is needed to elucidate their role in the “survival” of the CL during early pregnancy in cattle.

## 5. Conclusions

These data provide new evidence on the expression validation, using RT-qPCR, of RNA-Seq data for three randomly selected miRNAs in corpora lutea (CL) of pregnant cows versus regressed CL in non-pregnant cows. Of the three analyzed miRNAs, all showed the same trend of regulation in the sequencing data. However, only bta-miR-222-3p was downregulated; bta-miR-29c and bta-miR-2411-3p were not different. Some of the metabolic pathways enriched with genes targeted by the investigated miRNAs are FOXO, VEGF, and WNT signaling, and miRNAs in cancer. Gene ontology results show that 83% of the implicated protein classes act as specific transcriptional regulators for the genes targeted by the analyzed miRNAs.

## Figures and Tables

**Table 1:** The names, accession numbers, mature sequences, and chromosomes of miRNAs.

miR Name	Accession Number	Mature Sequence	Chromosome
bta-miR-222-3p	MIMAT0003530	AGCUACAUCUGGCUACUGGGU	chX
bta-miR-29c	MIMAT0003829	UAGCACCAUUUGAAAUCGGUUA	ch16
bta-miR-2411-3p	MIMAT0011973	GCUGAACUGUCUUACUCCCACAUCC	ch3

**Table 2:** The read counts and fold change regulations for the forty-four differentially regulated miRNAs from the re-analysis of RNA-Seq data in the comparison between corpora lutea (CL) from pregnant cows and regressed bovine CL. The miRNAs are listed in order of their adjusted *p*-value.

miR-ID	Normalized Read Counts	Log2 Fold Change of Pregnant CL vs. Regressed CL	Padj (Adjusted *p*-Value)
bta-miR-29c	117	1.98	2.23 × 10^−4^
bta-miR-21-5p	195,736	−1.59 *	2.29 × 10^−4^
bta-miR-27b	16,510	0.72	2.28 × 10^−4^
bta-miR-29a	7274	0.67	3.12 × 10^−4^
bta-miR-2332	1059	1.96	6.28 × 10^−4^
bta-miR-32	584	1.05	7.28 × 10^−4^
bta-miR-1248	227	1.99	7.56 × 10^−4^
bta-miR-2411-3p	662	1.91	8.56 × 10^−4^
bta-miR-652	331	1.21	7.56 × 10^−4^
bta-miR-150	66	−1.30	1.38 × 10^−3^
bta-miR-29b	340	1.52	1.67 × 10^−3^
bta-miR-222-3p	152	−1.53	3.06 × 10^−3^
bta-let-7i	26,531	−1.12	2.87 × 10^−3^
bta-miR-155	382	−1.27	2.87 × 10^−3^
bta-miR-677	74	1.79	2.91 × 10^−3^
bta-miR-199b	2357	−1.11	2.96 × 10^−3^
bta-miR-30a-5p	9908	0.86	2.96 × 10^−3^
bta-miR-33a	175	1.02	3.13 × 10^−3^
bta-miR-199a-3p	6340	−1.03	3.38 × 10^−3^
bta-miR-3604	2956	−1.05	4.33 × 10^−3^
bta-miR-3141	70	2.48	4.69 × 10^−3^
bta-miR-148b	910	0.65	6.21 × 10^−3^
bta-miR-2484	950	2.07	6.46 × 10^−3^
bta-miR-224	228	−0.98	1.12 × 10^−2^
bta-miR-146a	219	−1.01	1.25 × 10^−2^
bta-miR-23b-3p	1353	0.87	1.28 × 10^−2^
bta-miR-30b-5p	603	0.86	1.34 × 10^−2^
bta-miR-3600	28,676	0.92	1.34 × 10^−2^
bta-miR-126-5p	2018	0.89	1.46 × 10^−2^
bta-miR-22-5p	502	0.71	1.80 × 10^−2^
bta-miR-34a	211	0.94	1.98 × 10^−2^
bta-miR-379	1007	−1.28	1.98 × 10^−2^
bta-miR-149-5p	71	−1.03	2.08 × 10^−2^
bta-miR-214	472	−0.79	2.08 × 10^−2^
bta-miR-29e	57	1.15	2.24 × 10^−2^
bta-miR-339a	1409	0.82	2.24 × 10^−2^
bta-miR-365-3p	381	0.62	2.33 × 10^−2^
bta-miR-382	95	−1.59	2.37 × 10^−2^
bta-miR-146b	483	−1.21	2.48 × 10^−2^
bta-miR-2892	88	1.42	2.73 × 10^−2^
bta-miR-126-3p	3197	0.72	2.24 × 10^−2^
bta-miR-1246	105	1.52	4.58 × 10^−2^
bta-miR-411a	1555	−0.95	4.80 × 10^−2^
bta-miR-432	119	−1.44	4.82 × 10^−2^

* Negative value indicates the downregulation of bta-miR-222-3p in pregnancy compared to the regressed CL. Padj (adjusted *p*-value) for multiple comparisons.

## Data Availability

All data related to this research are presented in the research article.
